# Evidence for Paternal Leakage in Hybrid Periodical Cicadas (Hemiptera: *Magicicada* spp.)

**DOI:** 10.1371/journal.pone.0000892

**Published:** 2007-09-12

**Authors:** Kathryn M. Fontaine, John R. Cooley, Chris Simon

**Affiliations:** Department of Ecology and Evolutionary Biology, University of Connecticut, Storrs, Connecticut, United States of America; Centre National de la Recherche Scientifique, France

## Abstract

Mitochondrial inheritance is generally assumed to be maternal. However, there is increasing evidence of exceptions to this rule, especially in hybrid crosses. In these cases, mitochondria are also inherited paternally, so “paternal leakage” of mitochondria occurs. It is important to understand these exceptions better, since they potentially complicate or invalidate studies that make use of mitochondrial markers. We surveyed F_1_ offspring of experimental hybrid crosses of the 17-year periodical cicadas *Magicicada septendecim, M. septendecula,* and *M. cassini* for the presence of paternal mitochondrial markers at various times during development (1-day eggs; 3-, 6-, 9-week eggs; 16-month old 1^st^ and 2^nd^ instar nymphs). We found evidence of paternal leakage in both reciprocal hybrid crosses in all of these samples. The relative difficulty of detecting paternal mtDNA in the youngest eggs and ease of detecting leakage in older eggs and in nymphs suggests that paternal mitochondria proliferate as the eggs develop. Our data support recent theoretical predictions that paternal leakage may be more common than previously estimated.

## Introduction

Although mitochondrial DNA (mtDNA) exhibits a variety of inheritance patterns in eukaryotes [1], animal mitochondrial DNA is generally assumed to be maternally inherited. In the last 20 years, a few instances have been described in which animal mtDNA is transmitted through patrilines, a phenomenon termed “paternal leakage”. Paternal leakage challenges some of the assumptions involved in using mtDNA as a molecular or forensic marker [2]. Biparental mitochondrial inheritance followed by recombination can complicate phylogenetic reconstruction and molecular dating [3], [4]. Other authors [5], [6] note that divergence time estimates for *Drosophila simulans* and *D. mauritiana* differ fourfold, depending on whether an mtDNA polymorphism is ancient or the result of introgression between species.

Table 1 lists animal studies that demonstrate paternal leakage. Much of this work suggests that paternal leakage may be more likely when hybridization is involved, possibly due to the breakdown of mechanisms that normally destroy or exclude paternal mtDNA (for brief review, see [1]). Although reported cases of paternal leakage for animal mtDNA are few, the diversity of the taxa involved and the relative novelty of sensitive PCR-based detection techniques [7] combined with a lack of widespread effort to quantify this phenomenon and the inability of researchers to detect hybridization if maternal and paternal mitochondrial genotypes are identical, raise the possibility that paternal leakage may be more widespread than once thought. Here we present evidence of paternal leakage in hybrid crosses involving three species of 17-year periodical cicada, *Magicicada septendecim, M. septendecula* and *M. cassini*.

**Table 1:** Some examples of paternal leakage in the literature.

Common Name		Reference
**Heterospecific crosses**		
Silkmoth	*Antheraea pernyi X A. roylei*	[49]
Fruit fly	*Drosophila mauritiana X. D. simulans*	[42]
Fruit Flies	*Drosophila mauritiana X. D. simulans*	[44]
Tobacco budworm	*Heliothis virescens X H. subflexa*	[31]
Periodical Cicada	*Magicicada septendecim X M. cassini*	This study
Periodical Cicada	*Magicicada septendecim X M. septendecula*	This study
House Mouse	*Mus musculus X M. spretus*	[50]
House Mouse	*Mus musculus X M. spretus*	[38]
House Mouse	*Mus musculus X M. spretus*	[51]
**Conspecific crosses**		
Honeybee	*Apis mellifera carnica X A. mellifera capensis*	[33]
Cow	*Bos taurus*	[52]
Scorpion	*Buthus mardoechi*	[53]
Frillneck lizard	*Chlamydosaurus kingii*	[54]
Fruit Flies	*Drosophila mauritiana X. D. simulans*	[44]
Anchovy	*Engraulis encrasicolus*	[55]
Human	*Homo sapiens*	[56]
Scorpion	*Mesobuthus caucasius*	[53]
Scorpion	*Mesobuthus eupeus*	[53]
Scorpion	*Mesobuthus gibbosus*	[53]
Sheep	*Ovis aries*	[57]
Eastern tiger swallowtail	*Papilio glaucus*	[58]
Great tit	*Parus major major X P. major minor*	[59]
Flatfish	*Platichthys flesus*	[60]

### Background

Paternal leakage is of particular interest in the periodical cicadas of North America (Hemiptera: *Magicicada* spp.) because mitochondrial markers have been central in evolutionary studies of these species. For example, an abrupt mtDNA haplotype (and nuclear color polymorphism) transition has been interpreted as evidence for a lack of gene flow between courtship-song-displaced, synchronic species [8]–[10], and the same haplotype boundary has been interpreted as evidence for sex-biased dispersal [11]–[13]. Either of these interpretations would be complicated by paternal leakage.

The seven currently-recognized 13- and 17-year periodical cicada species (*Magicicada septendecim* {17}, *M. tredecim* {13}, *M. neotredecim* {13}, *M. cassini* {17}, *M. tredecassini* {13}, *M. septendecula* {17}, and *M. tredecula* {13}), belong to three species groups (-decim, -cassini, and -decula), and each species is most closely related to one with the alternative life cycle (13 or 17 years), suggesting multiple allochronic speciation events. Within each *Magicicada* species group, mitochondrial genetic differences are slight (0% between 13- and 17-year -decula or -cassini species pairs and 2.6% uncorrected between the *M. septendecim/M. neotredecim* and *M. tredecim*). Uncorrected mtDNA distances are 3–4% when comparing -decula to -cassini species and 7–8% between members of either of these species groups and the -decim group [14].

Within a given geographical region, periodical cicadas emerge synchronously in mass numbers, and adults form mixed-species choruses. Different regions are on different emergence schedules with the “brood” year designated by sequential Roman numerals. Although choruses provide opportunities for hybridization, mixed-species matings are rare [15]–[19]. More detailed background information on periodical cicada broods, species, and behavior is available elsewhere [10], [14], [16], [20]–[24].

After emerging from the ground, periodical cicada nymphs undergo ecdysis immediately, after which they spend 5–9 days as relatively inactive, teneral adults [25]–[28]. After the teneral period, adults become more active and mate. Because periodical cicadas are superabundant, unmated teneral adults are relatively easy to obtain. Although these insects are difficult to maintain in the laboratory, they may be maintained and manipulated under semi-natural conditions in outdoor cages containing living, woody vegetation. When males and females are confined in cages and given no choice of mates, they will engage in hybrid matings, and hybrid eggs and nymphs are viable [16], [29].

We developed a PCR-based method that makes use of specific primers and known *Magicicada* haplotype differences to detect rare mtDNA haplotypes in experimentally-crossed cicadas (our method is similar to that in [30]). We tested this method on mtDNA mixtures made by combining, in different proportions, the DNA of wild-caught individuals of known species, and we then used these primers to investigate the possibility of paternal leakage in reciprocal crosses of *M. septendecim* with *M. cassini* and *M. septendecula*.

## Results

### Paternal Leakage

We were unable to extract DNA from one pooled sample of 6-week old eggs, and two extractions of 16-month nymphs from *M. septendecim* homospecific crosses failed to amplify. All other extractions and amplifications were successful. Paternal mtDNA was not found in the 1-day old hybrid eggs, but it was found in all older age groups (4 days, 3 weeks, 6 weeks, 9 weeks, and 16 months; Table 2). The single 4-day old sample was present because a new eggnest from that particular female had to be re-cut because the day-old nest originally cut and dissected was found to be empty. As noted by White [29], heterospecific crosses involving male -decim were fewer in number than other heterospecific crosses because female-cassini and -decula are less likely to mate with the less aggressive -decim males.

**Table 2:** Number of crosses showing amplification of maternal and paternal haplotypes.*

	1 Day*		3 Weeks		6 Weeks		9 Weeks		16 Months		Total	Total
	mat	both	mat	both	mat	both	mat	both	mat	both	mat	both
cassini M×decim F	10	1	2	12	5	8	0	4	5	10	22	35
decula M×decim F	8	0	9	3	6	3	-	-	-	-	23	6
decim M×cassini F	3	0	0	1	-	-	4	1	-	-	7	2
decim M×decula F	-	-	0	1	0	1	-	-	-	-	0	2
decim×decim	-	-	-	-	-	-	5	U	13	U	18	U
cassini×cassini	-	-	-	-	-	-	3	U	-	-	3	U
total	21	1	11	17	11	12	12	5	18	10	73	45

* “mat” tested positive for maternal mtDNA only; “both” tested positive for both maternal and paternal mtDNA; “U” paternal inheritance cannot be determined because the paternal genome is identical to the maternal; “-” no eggs or nymphs were collected for this cross at this time period or samples that were collected failed to extract/amplify (only one pooled sample of 6-week old eggs failed to extract–a -decula M×-decim F; only two extractions failed to amplify and both of these were -decim M×-decim F 16-month old nymphs). Fewer offspring from crosses involving -decim males and heterospecific females were sampled because -decula and -cassini females most often rejected -decim males.

### Ruling Out Numts

To ensure that the primers were amplifying mitochondrial COI and not nonfunctional nuclear copies of mitochondrial genes (NUMTs), the amplified paternal and maternal mtDNA from representative sequences of hybrid mixed-haplotype eggnest extractions (8 sequences from -decim×-cassini and 8 from -decim×-decula) were compared to the original COI sequence. All of the sequences exactly corresponded to the original COI sequence of the proper primer set and none of the species-specific primer sets produced sequence that had errors (subpeaks, weak sequence, etc.) that suggested multiple templates. Furthermore, we found no stop codons in any of the sequences, and all species-specific base substitutions were silent (e.g., did not affect amino acid sequence), suggesting that our sequences belong to functional genes.

### Ruling Out Contamination

We were careful to avoid contamination that would lead to false positive results (i.e., the detection of mtDNA from heterospecific contamination rather than from paternal leakage). The best evidence against heterospecific contamination was that sample DNA from homospecific crosses amplified with homospecific primers but never with heterospecific primers. Homospecific contamination would be undetectable because contaminant DNA would be identical to sample DNA; however, this would not change our current conclusions about paternal leakage because we cannot detect paternal mtDNA in homospecific crosses for the same reason.

Contaminant DNA is most likely to be amplified when there is little or no target DNA. Every set of PCR reactions (made from a single reaction mix) included a negative control (no maternal or paternal target mtDNA) and none of these control reactions ever showed any sign of amplified DNA.

**Figure 1:**
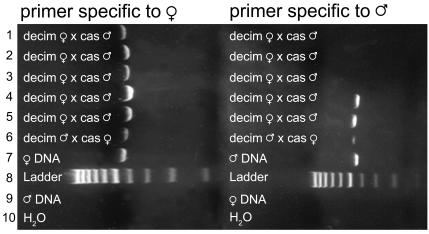
Example of -decim×-cassini hybrid PCR product in 2% agarose gel stained with Sybrsafe. First column: primer specific to maternal mtDNA; Lanes 1-5: -decim F×-cassini M; Lane 6: -decim M×-cassini F; Lane 7: Maternal species DNA; Lane 8: 100 bp Ladder; Lane 9: Paternal species DNA; Lane 10: H_2_0 negative control (No DNA); Second column: primer specific to paternal mtDNA. Lanes 1-5: -decim F×-cassini M; Lane 6: -decim M×-cassini F; Lane 7: Paternal species DNA; Lane 8: Ladder; Lane 9: Maternal species DNA; Lane 10: H_2_0 negative control (No DNA). Lanes 4-6 show paternal leakage.

**Figure 2:**
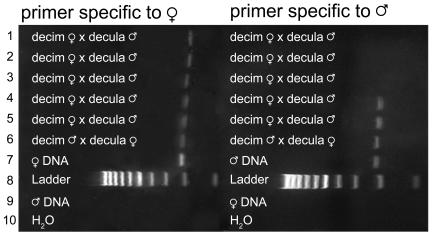
Example of -decim×–decula hybrid PCR product in 2% agarose gel stained with Sybrsafe. First column: primer specific to maternal mtDNA; Lanes 1-5: -decim F×-decula M; Lane 6: -decim M×-decula F; Lane 7: Maternal species DNA; Lane 8: Ladder; Lane 9: Paternal species DNA; Lane 10: H_2_0 negative control (No DNA); Second column: primer specific to paternal mtDNA. Lanes 1-5: -decim F×-decula M; Lane 6: -decim M×-decula F; Lane 7: Paternal species DNA; Lane 8: Ladder; Lane 9: Maternal species DNA; Lane 10: H_2_0 negative control (No DNA). Lanes 4-6 show paternal leakage.

A final pair of controls included DNA from non-hybrid adults (extracted from a single leg) of both species involved in each cross. This adult DNA was tested with both homo- and hetero-specific primers. These controls were included in every PCR set (made from a single batch of reaction mix) and were visualized on the same gels as the experimental samples (Figs. 1–2). Failure of heterospecific primers and success of the homospecific primers assured us that the correct primers had been added to all reaction mixtures and that contamination was not present.

## Discussion

Our results suggest that paternal leakage occurs in hybrid *Magicicada*. Until recently, the most sensitive techniques for detecting paternal leakage involved backcrossing experiments [31] that could not be used to detect leakage in wild populations or in animals (such as periodical cicadas) with long life cycles. Although PCR-based methods may be susceptible to NUMTs (nonfunctional nuclear copies), whose transmission is biparental, for NUMTs to explain our results each periodical cicada species would need to have an exclusive NUMT not found in the other species, and this exclusive NUMT would need to match the paternal mitochondrial sequence of each cross exactly. We consider this possibility to be highly unlikely. In addition, as explained in the results section, contamination controls argue in favor of paternal leakage.

Our results suggest that paternally-transmitted mitochondria in *Magicicada* proliferate during development. We detected paternal leakage in all age groups examined except 1-day old eggs. The relative difficulty of detecting leakage in the youngest eggs suggests a scenario in which, as expected, paternal mitochondria are present but extremely rare (and difficult to detect) at first. They then proliferate as the eggs develop (and thus they become more reliably detectable). Our results cannot be explained by ejaculate residue contamination during oviposition; if we were detecting surface contamination rather than leakage, we would expect that paternal haplotypes might be detected in some eggs, but undetectable in hatched nymphs. Given the strong possibility that paternal mitochondria are replicating (if so, then template mtDNA concentrations changed throughout our study), it is not advisable to use the results of these experiments to evaluate the relative frequencies of paternal leakage of the different crosses, since quantitative conclusions from our experiments may be confounded by changing mtDNA template concentrations.

Although we detected paternal mtDNA in hybrid juvenile periodical cicadas, it remains unknown whether paternal mtDNA will persist through development or whether it enters the germ line [32]. In at least one example from holometabolous insects (those with complex metamorphosis), heteroplasmy is not maintained. Meusel [33] found that in honeybees, the paternal contributions disappeared during development. We suspect that the evidence for proliferation of paternal mtDNA in our developing cicadas and the simple metamorphosis of cicadas make it likely that heteroplasmy will persist through to adulthood. We have left some nymphs from this study growing underground and we are continuing to monitor them for evidence of heteroplasmy.

Several factors are thought to contribute to the rarity of paternal leakage. First, in some cases, sperm may not enter oocytes (observed in some tunicates), or sperm may not contain mitochondria (observed in some crayfish species [34]), so paternal leakage is not possible. Even in organisms in which sperm enter oocytes and sperm contain mitochondria, maternal mtDNA outnumber paternal mtDNA by as much as 10,000 fold [35], and the relatively low numbers of paternal relative to maternal mitochondria may have a swamping effect when a zygote is formed [36], [37]. However, one common explanation for the rarity of paternal leakage is that oocytes have mechanisms for actively destroying objects with foreign surface proteins, or, as shown in mammals, that paternal mitochondria are ubiquitinated (either during spermatogenesis or after fertilization) and destroyed by the oocyte [38]–[41].

Some studies of *Drosophila* [42], [43] suggest that leakage is most likely if the genetic difference between species is approximately 2.5% or greater due to the fact that oocyte enzymes cannot recognize and destroy distantly-related sperm mitochondria, but a more recent study of *Drosophila* [44] demonstrates paternal leakage between closely related subspecies (<2.5% difference). The *Magicicada* species used in our experiments exhibit roughly 7–8% sequence divergence (uncorrected) but other *Magicicada* species pairs are more closely related (3–4% for the -cassini versus -decula siblings, 2.6% for *M. septendecim* vs. *M. tredecim*, and close to 0% for *M. cassini* vs. *M. tredecassini* and *M. sependecula* vs. *M. tredecula*) [14] suggesting the opportunity for further tests of this hypothesis.

Paternal leakage may be more common than previously thought for several reasons. In conspecific crosses paternal mtDNA may be undetectable if mtDNA haplotype variation within populations or among interbreeding populations is slight or absent as in many animal mtDNA studies (e.g., animal species living in previously glaciated areas of North America and Europe [45]), leading to biases against detecting leakage except in cases involving hybridization [46]. Other reasons that paternal leakage may be difficult to detect are that it may occur in some individuals and not others, or with some kinds of crosses and not others. Kondo *et al*. [42] found that of 331 lines of *Drosophila simulans* backcrossed with *D. mauritiana* (backcrossed for ten generations), only four lines showed evidence for paternal leakage of mtDNA. Significantly, in three of these four lines, the maternal mtDNA was completely replaced by the paternal mtDNA while in the fourth, individuals were heteroplasmic. All of these crosses were *D. simulans* females crossed with *D. mauritiana* males. In other hybrid crosses, it has been shown that mtDNA from one of the parental species may not survive as well as the other in a hybrid background [5]. Finally, paternal leakage may have gone unnoticed because researchers, expecting it to be virtually non-existent, have not looked for it; evidence for heteroplasmy in mtDNA sequences may have been taken to be artifacts or low-level nuclear copies of mtDNA.

Other evidence that paternal leakage of animal mtDNA might be surprisingly common is presented by Piganeau *et al*. [4], who found strong evidence of mtDNA recombination (between presumably maternal and paternal mtDNA) based on statistical analysis of 279 animal taxa (156 vertebrates, 57 arthropods, 29 mollusks, 12 nematodes, and 11 echinoderms). Their analyses did not allow them to pinpoint the exact taxa that displayed recombination but they were able to isolate the twenty species that contributed most to the result and were therefore most likely to contain recombinant genotypes. These twenty animal taxa comprised a wide taxonomic sampling (one nematode, one insect, one collembolan, one crustacean, one cephalopod and 13 vertebrates). Two bivalve mollusks were also represented but these species are known to have regular, tissue-specific, double uniparental inheritance of mtDNA. There was no indication that the frequency of recombination varied across taxonomic groups. In two of the twenty strongest cases, recombinant individuals could be recognized and both cases appeared to involve hybridization between subspecies.

Our data add to the growing number of successful interspecific paternal leakage studies. We suggest the need for more surveys of natural populations of hybrid individuals and for more experimental crosses between species and between divergent haplotypes within species to look for paternal leakage. Such studies are important for clarifying potential problems with analyses that rely on exclusively maternal mtDNA inheritance. In addition, such studies might help clarify the reasons why mitochondrial inheritance is ever uniparental.

## Materials and Methods

**Figure 3:**
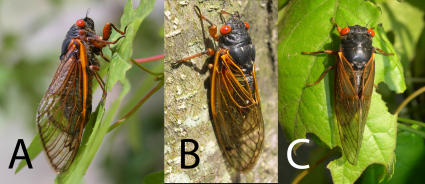
Research organisms and experimental set up. (A) *Magicicada septendecim* female (Brood X), (B) *Magicicada cassini* female (Brood X), (C) *Magicicada septendecula* male (Brood IX). Photographs in Figs. 3–7 by C. Simon.

During the emergences of Brood IX (2003) and X (2004) of 17-year periodical cicadas, we collected unmated (newly emerged) cicadas from various locations (Table 3) and performed purebred and cross-species matings by enclosing males and females in small cages (Figures 3–7; crosses performed and numbers of matings are reported in Table 4). Mating cages contained either males and females of the same species (controls) or males of one species with females of another species (heterospecific crosses) so individuals were not free to choose the species with which they mated. Natural hybridization is rare, partly because females are unresponsive to the songs of heterospecifics [47] (Typical songs from each species group are included in Audio S1, S2, S3, S4). We facilitated hybrid matings by placing heterospecific mating cages near homospecific cages. This arrangement allowed females to hear males of their own species and to signal sexual receptivity, increasing the odds that a heterospecific male in her own cage would mate her. After mating, females were isolated in individually marked cages surrounding live tree branches suitable for oviposition and feeding. The cages were monitored for oviposition, and at four time periods after laying (1 day, 3 weeks, 6 weeks and 9 weeks), approximately 3 eggnests (approximately 60 eggs) were collected from each female's cage. After the eggnests were cut from the branches, the eggs were removed and stored in 100% ethanol. We found that one of the eggnests dissected from the 1-day age group was empty, so we cut and dissected another eggnest from the same female; by the time we did this, the eggnest was 4 days old. All remaining eggnests were clipped from the trees just prior to hatching and the hatching nymphs were allowed to burrow into the ground in marked 1 m^2^ plots in a second-growth Oak-Hickory forest in Connecticut. After approximately 16 months, cicada nymphs from one control and one hybrid cross (-decim×-decim and -cassini male×-decim female) were excavated and stored in 100% ethanol. All females in this experiment were permitted to mate only once, ruling out mixed paternity among the eggs of an eggnest.

**Figure 4 pone-0000892-g004:**
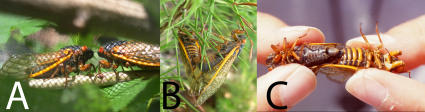
Courtship and mating in periodical cicadas. (A) A pair of *M. septendecim* courting inside a screen mesh cage, (B) A pair of *M. septendecim* mating (Brood IX), (C) A *M. septendecim* female/*M. cassini* male mating pair.

**Figure 5 pone-0000892-g005:**
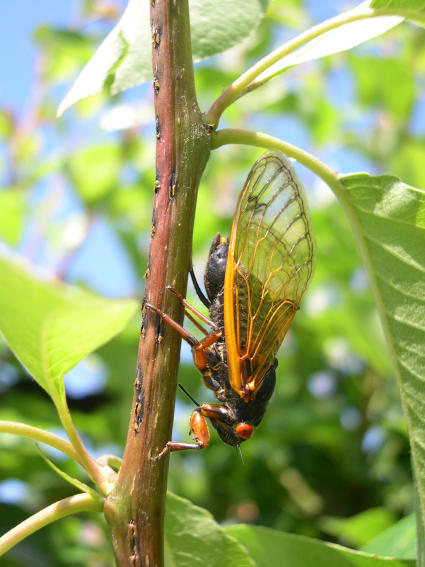
* M. cassini* female ovipositing; note additional eggnest scars on twig.

**Figure 6 pone-0000892-g006:**
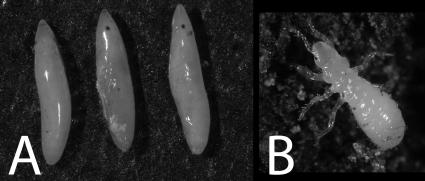
(A) Three *Magicicada* eggs removed from an eggnest, (B) A first instar *Magicicada* nymph.

**Figure 7:**
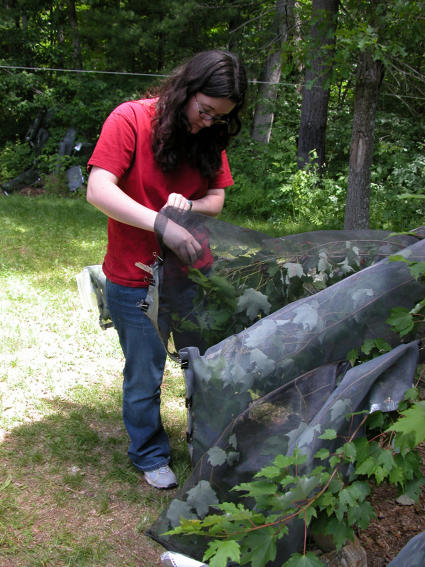
K. Fontaine inspecting a mating cage for mating pairs.

**Table 3:** Periodical cicada collection sites.

Brood	Species	Collection Sites
XI	*M. septendecim*	Wilkes County, NC; Pipestem State Park, VA
	*M. cassini*	South Gap, VA; Bluestone State Park, VA; Vernick Creek, VA
	*M. septendecula*	Bluestone State Park, VA
X	*M. septendecim*	Hunterdon County, NJ (Princeton Area)
	*M. cassini*	Hunterdon County, NJ (Princeton Area)

**Table 4:** Percentage of caged females that mated for each cross in the 2003 Brood IX and 2004 Brood X experiments.*

2003 Brood IX	-decim male	-cassini male	-decula male
-decim female	-	48.50%	30.90%
-cassini female	10.90%	-	-
-decula female	5%	-	-
2004 Brood X			
-decim female	73.30%	61.50%	
-cassini female	33.30%	54.60%	
-decula female	-	-	

* An (-) indicates that that cross was not performed. Better weather may account for the increased mating frequency in Brood X when compared to Brood IX.

DNA was extracted from legs of adult cicadas belonging to the three different *Magicicada* species groups using the Nucleospin Tissue kit (BD Biosciences Clontech; Palo Alto, CA) following instructions provided by the manufacturer. Extractions were PCR amplified using primers C1-J-2195 and TL2-N-3014 for 30 cycles [48]. PCR product was cleaned and sequenced using BigDye terminator chemistry and an ABI Prism 3100 capillary sequencer. On the basis of these sequences, we developed internal 25-mer COI primers (Table 5) with 3′ ends that anneal to polymorphisms unique to each species group. When annealing temperatures were set to 58° (-decim×–decula crosses) or 60° (-decim×–cassini crosses) these species-specific primers successfully amplified DNA from the appropriate species and failed to amplify heterospecific DNA (Figs. 1–2). To ensure that the primers were amplifying the desired mtDNA segment, the size of the PCR product was estimated with a DNA ladder (exACTGene 100 bp DNA Ladder; Fisher Scientific; Pittsburgh, PA). The PCR product was also sequenced and compared to the COI sequence that was used to develop the primers.

**Table 5:** Species-specific primer sequences*.

Primer	Species	Sequence (5′-3′)
C1-J-2195	Universal	TTGATTTTTTGGTCATCCAGAAGT
TL2-N-3014	Universal	TCCAATGCACTAATCTGCCATATTA
C1-J-2287	-decim	GAATCATTTGGATCATTAGGAATGA
	-cassini	GAATCTTTTGGGTCACTAGGAATAG
C1-N-2712	-decim	AAAGAAGGTTAAATTTACCCCAAT
	-cassini	GAAAAAAGTTAAATTTACTCCAAC
C1-N-2787	-decula	TCTTCTTCCAATAGAAGACATAATA
	-decim	TCTTCTTCCAATAGAAGATACAATG
C1-J-2607	-decula	AGGTGCAGTGTTTGCAATCTTGGGG
	-decim	AGGTGCAGTATTTGCAATTTTAGGA

* differences between the sequences are underlined.

**Figure 8:**
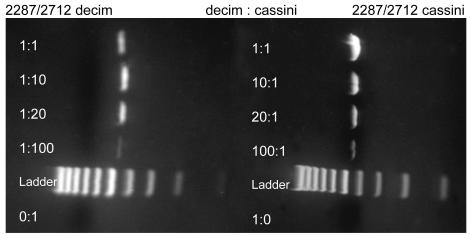
Dilution test with different volume ratios of experimentally mixed -decim:-cassini DNA in a 2% agarose gel stained with Sybrsafe. First column: Amplification with -decim-specific primers 2287/2712; Second column: Amplification with -cassini-specific primers 2287/2712. The less abundant mtDNA type was revealed using species-specific primers. Ladder is a 100 bp ladder with 1000 bp band on left.

**Figure 9:**
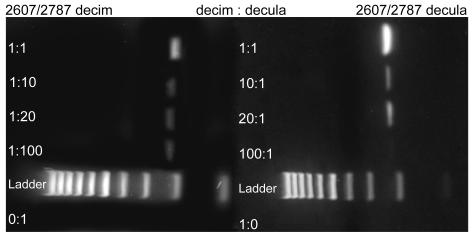
Dilution test with different volume ratios of experimentally mixed -decim:-decula in a 2% agarose gel stained with Sybrsafe. First column: Amplification with -decim-specific primers 2607/2787; Second column: Amplification with -decula-specific primers 2607/2787. The less abundant mtDNA type was revealed using species-specific primers. Ladder is a 100 bp ladder with 1000 bp band on left.

The sensitivity of the diagnostic primers was tested on mixed DNA samples containing DNA from two species in the following ratios: 1∶1, 1∶10, 1∶20, 1∶100, 0∶1, 10∶1, 20∶1 and 100∶1, 1∶0. All mixtures were PCR-tested for the both DNA types with the species-specific primers. Three of the four species-specific primer sets were able to detect the less abundant DNA up to the 1∶100 dilution level (Figs. 8–9). The -decula-specific primers could detect -decula mtDNA up to the 1∶20 dilution level.

From each eggnest, we pooled 10 fertilized eggs and extracted their DNA; nymphs were extracted singly, and all extractions were performed as above. We probed all collected eggs and nymphs with species-specific primers to detect the presence of both maternal and paternal mtDNA haplotypes. Sample sizes for amplified nymph and pooled-egg samples are listed in Table 2. Each PCR reaction included a negative species control (PCR reaction with heterospecific DNA template from an adult), positive species control (conspecific DNA template from an adult) and negative PCR control (dH_2_0, no DNA but all other reagents).

## Supporting Information

Audio S1 Calling song of *M. septendecim.*
(0.13 MB WAV)

Audio S2 Calling song of *M. cassini.*
(0.17 MB WAV)

Audio S3 Calling song of *M. septendecula.*
(0.30 MB WAV)

Audio S4 A *Magicicada* chorus containing *M. septendecim, M. cassini,* and *M. septendecula.*
(4.30 MB WAV)
